# The current status of crop improvement using DNA marker technology

**DOI:** 10.1270/jsbbs.76.1

**Published:** 2026-03

**Authors:** Toshio Yamamoto, Hiroyuki Fukuoka

**Affiliations:** 1 Institute of Plant Science and Resources, Okayama University; 2 Plant Breeding and Experiment Station, Takii & Company, Limited

The rice whole-genome sequencing project was a highly ambitious undertaking at the time of its inception. However, through internationally coordinated efforts in Japan and rapid technological progress, the project was successfully completed 20 years ago (IRGSP 2005). This milestone has significantly advanced agricultural science in two major directions: fundamental research on gene isolation and functional analysis in plants; and the application of DNA markers in breeding. Nevertheless, the ultimate goal, which is developing innovative varieties on demand based on a comprehensive understanding of agricultural traits, remains a work in progress. While the project initially promised a bright future for agriculture, the realization of this vision has proven considerably more complex than originally anticipated. As our understanding of traits deepens, new challenges continue to emerge.

The most direct application of crop genome information is in breeding, where DNA markers, which are genomic markers that leverage nucleotide sequence polymorphisms, serve as primary technical tools. DNA marker-assisted selection (MAS) offers numerous advantages, including independence from environmental influences and breeder expertise during phenotypic evaluation, low cost, and flexible selection timing based solely on DNA availability. Prior to the availability of genome sequences, developing even a single DNA marker required considerable effort. However, in this era where even the wheat genome, which is 40 times larger than the rice genome, has been sequenced, DNA marker development has become increasingly streamlined. Studies on MAS and its practical implementation in breeding programs are now routinely conducted across various crop species. Furthermore, continuous advancements in genomics-related technologies are expanding both the scope and applications of these tools. Consequently, a comprehensive review of the current state of DNA marker-assisted crop breeding is highly significant for both researchers and breeders.

This special issue summarizes case studies demonstrating the successful application of DNA markers in breeding selection, focusing primarily on major crops cultivated in Japan. Specifically, it covers the identified gene loci that have been successfully implemented in breeding programs, the impact of the resulting improved varieties, the practical gains in breeding efficiency achieved through this technology, and prospects for future genome-related technologies. This overview is provided by experts with both practical breeding experience and a deep understanding of genomic information. Goto *et al.* (2026) present the current state of genomic breeding in rice, which is a model crop for many species. Kato *et al.* (2026) describe examples of practically applied agronomic trait genes in soybean. This is followed by Tanaka *et al.* (2026) who introduce challenges confronting genomic breeding in wheat. Nunome *et al.* (2026) review useful agronomic traits in several vegetable crops and in strawberries, classified as a vegetable crop in Japanese agricultural science, along with the corresponding DNA markers for their selection. Ishiguro *et al.* (2026) present a cross-disciplinary approach that integrates tea genome analysis with cultivation practices. We also present perspectives on the philosophical and methodological aspects of genomic breeding, drawing on a recent case study of tomato disease resistance breeding at a global seed company, as described by Verweij *et al.* (2026). Additionally, we include two research papers focused on citrus genomic breeding: one describes an efficient haplotype imputation system across genotyping platforms (Shimizu and Nonaka 2026), and another discusses the identification of markers to distinguish commercially valuable varieties derived through mutagenesis (Ishii *et al.* 2026).

We extend our sincere gratitude to all authors and anonymous reviewers who contributed to this special issue, providing valuable insights that will undoubtedly advance the field. Finally, we gratefully acknowledge the Editor-in-Chief, Dr. Izawa, for granting us the opportunity to address this special issue.
